# Terrestrial Laser Scanning of Lunar Soil Simulants

**DOI:** 10.3390/ma15248773

**Published:** 2022-12-08

**Authors:** Marzena Damięcka-Suchocka, Jacek Katzer

**Affiliations:** 1 Faculty of Civil Engineering, Environmental and Geodetic Sciences, Koszalin University of Technology, Śniadeckich 2, 75-453 Koszalin, Poland; 2 Faculty of Geoengineering, University of Warmia and Mazury in Olsztyn, Prawocheńskiego 15, 10-720 Olsztyn, Poland

**Keywords:** lunar soil simulant, terrestrial laser scanning, Moon, *intensity*, spectrometer

## Abstract

In the near future, permanent human settlements on the Moon will become increasingly realistic. It is very likely that the Moon will serve as a transit point for deep space exploration (e.g., to Mars). The key to human presence on the Moon is the ability to erect the necessary structures and habitats using locally available materials, such as lunar soil. This study explores the feasibility of using terrestrial laser scanning technology as a measurement method for civil engineering applications on the Moon. Three lunar soil simulants representing highland regions (LHS-1, AGK-2010, CHENOBI) and three lunar soil simulants representing mare regions (LMS-1, JSC-1A, OPRL2N) were used in this study. Measurements were performed using three terrestrial laser scanners (Z+F IMAGER 5016, FARO Focus^3D^, and Leica ScanStation C10). The research programme focused on the radiometric analysis of datasets from the measurement of lunar soil simulants. The advantages and limitations of terrestrial laser scanning technology for possible lunar applications are discussed. Modifications of terrestrial laser scanners that are necessary to enable their use on the Moon are suggested.

## 1. Introduction

During the last decade, many countries (e.g., the United States, Russia, China, Japan, India, Israel, and the European Union) have seen a noticeable increase in interest in space exploration by public and private institutions. Currently, the natural satellite of Earth is the first and main target for human space research and possible exploration. It has a unique location (a relatively small distance from Earth of approximately 375,000 km); a wealth of raw materials such as iron, aluminium, silicon, helium3, and hydrogen; and almost non-existent geological activity [1]. Many studies have reported that water (frozen in deep and dark polar craters) is also present on the Moon [2]. In practice, a sustainable human presence on the Moon will require the construction of multiple structures, such as habitats for crew, various technical buildings, landing pads, and roads for lunar rovers [3]. The most important characteristic of future lunar construction efforts will be fully harnessing in situ resource utilisation (ISRU) [4,5].

The surface of the Moon is covered with a layer of fine-grained material (the regolith) with an average thickness of 4–5 m and 10–15 m in the mare and highland regions, respectively [1]. In some locations, the thickness of the regolith layer can reach 100 m [6,7]. To date, approximately 115 kg of lunar regolith from Apollo missions [8], 0.3 kg from Luna missions [8], and 1.7 kg from Change’e-5 missions [9] have been transported to Earth. Many researchers in various fields are interested in this material; however, owing to the small amount that is accessible, it is available only for selected small-scale research studies. The scarcity of lunar regolith for scientific research has forced scientists to use lunar soil simulants (LSSs) as proxy materials for real lunar regolith. Therefore, dozens of different LSSs have been developed over the years by various universities, institutes, and research entities. However, only a few of these are commercially available. The LSS should reflect all or selected properties, e.g., physical, mineralogical, or chemical, of real lunar regolith as closely as possible [10,11]. To date, scientists have conducted large-scale lunar research programs using LSSs in many areas, such as the preparation of components for the construction of habitats [12,13], growing crops [14,15], testing landers, and analysing the wheel–soil interactions of lunar rovers [16,17]. Future civil engineering tasks associated with erecting lunar structures will require contactless, accurate, and fast measurement technologies. In the authors’ opinion, terrestrial laser scanning (TLS) meets all of the key requirements for such applications.

Terrestrial laser scanning technology is one of the fastest and most-efficient measurement methods (two million points/s). TLS measurements also have high accuracy. The working principle of a terrestrial laser scanner is the emission of a laser beam by a transmitter. The beam is deflected in the vertical (*α*) and horizontal (*β*) directions by a system of rotating mirrors [18]. When the laser beam strikes the surface of an object, it is reflected, and part of the beam’s energy returns to the receiver. The distance (*R*) between the scanner and the target can then be determined using time-of-flight (TOF) or phase-shift (PS) distance measurement methods. Based on *α*, *β*, and *R*, the three-dimensional point positions (*X*, *Y*, *Z*) can be calculated in the local coordinate system. Additionally, a terrestrial laser scanner registers the power of the backscattered laser beam, which depends on the physicochemical properties of the scanned materials, such as the colour, roughness, and saturation [19]. The power of the returned laser beam has a significant effect on the maximum distance of the TLS measurement and its general precision. The value of the *intensity* (the ratio between the emitted and reflected powers of the laser beam) is commonly used in scientific research to assess the different properties of scanned materials [20]. It has been demonstrated that TLS is suitable for various tasks associated with civil engineering [21], environmental protection [22], documentation of cultural heritage [23], and industrial applications [24]. Taking all the above facts into account, the authors decided to test and assess the efficiency of TLS for future construction efforts on the Moon.

One of the proposed methods for building an initial lunar base on the Moon is an inflatable composite habitat structure that will be covered with lunar soil [25] or 3D-printed building blocks [26]. TLS technology could be used to prepare the building site for a habitat, during the process of erection, and for final checks before commissioning the habitat for use. In addition, TLS technology can be utilised for lunar cave measurements and data collection for creating digital terrain models. It should be noted that the utilisation of natural caves and lava tubes on the Moon could provide a natural shelter for future colonists [27,28]. Such solutions may provide living quarters with some protection against solar and gamma radiation, meteoroid impacts, and large temperature fluctuations (from −183 °C to +106 °C).

In the current study, the authors conducted a thorough and successful research programme dedicated to the measurement of LSSs using terrestrial laser scanners. Six LSSs and three scanners were used during the research programme. The tests focused on the assessment of TLS technology for possible future lunar applications for a variety of civil engineering tasks. The main aim of this study was the radiometric analysis (*intensity*) of TLS point clouds from the measurement of LSSs. Initial research in this area was reported in [29,30]. In these studies, only ilmenite was used as the LSS. Therefore, more detailed research was needed using a larger number of LSSs that are recognised by the scientific community and commonly used for various lunar research tasks. In this study, the advantages and limitations of using TLS technology for lunar construction applications are discussed. Modifications that should be made to the scanners to cope with the harsh lunar environment are suggested. Laser measurements of the Moon have already been performed by the Lunar Orbiter Laser Altimeter (LOLA) [31]. The main aim of LOLA large-scale measurements was to determine the shape of the Moon and to prepare geodetic topographic maps. Small-scale laser measurements in the context of future lunar civil engineering projects have not been carried out so far. Thus, the novelty of the present study is that it represents the first time that regular TLS scans of LSSs have been conducted. Moreover, during the research programme, six lunar soil simulants were used. All simulants in question are thoroughly described in the literature and were developed by scientific institutions for particular research purposes. All the simulants have been used globally by numerous research teams for different scientific tests. Taking into account that three scanners were used for the scanning of LSSs, a unique set of results was obtained. This original approach enabled thorough analysis of the feasibility and limitations of future TLS on the Moon.

## 2. Materials and Equipment

**Figure 1:**
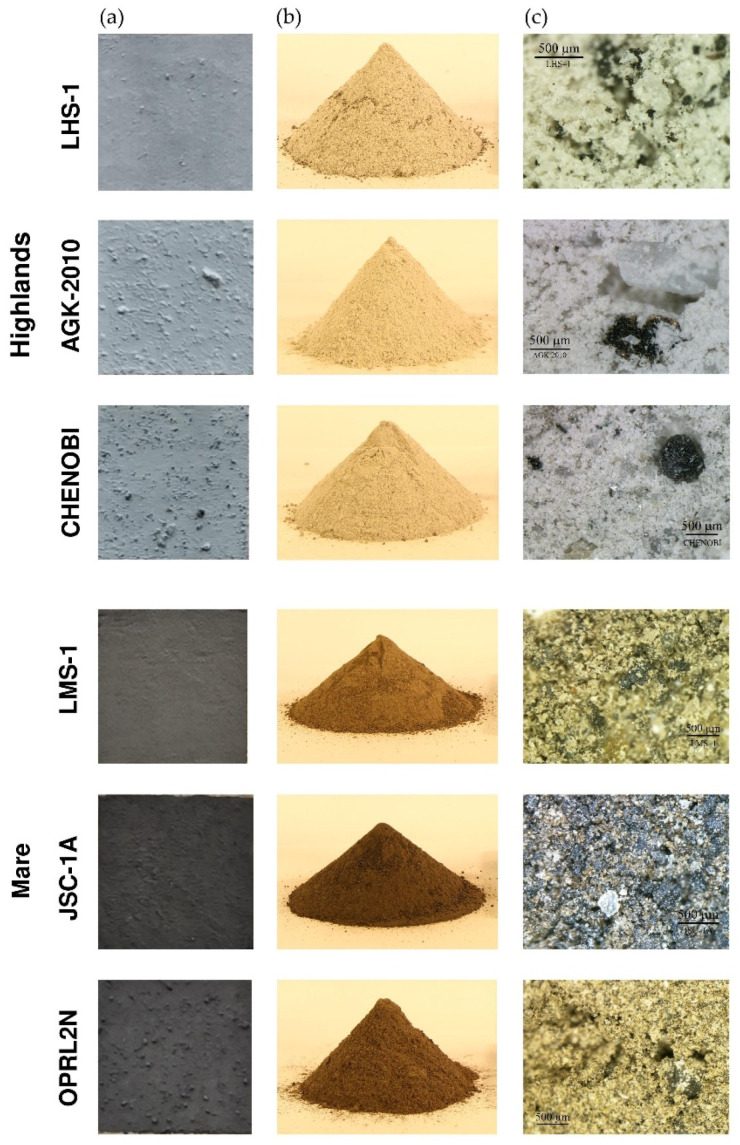
LSSs: prepared targets (**a**), piles of raw materials (**b**), and microscopic views (**c**).

The authors used six LSSs (previously described in the literature) for laser measurements. Considering that future lunar structures will be located in different places on the Moon (highlands or mare regions), the LSSs used represented both regions. The highlands and mare regions are characterised by different mineral compositions of the regolith. The mare regions are richer in iron and titanium; therefore, they are darker than the highland regions, which consist of lighter silicate minerals [1]. The LSSs used in this study are listed in Table 1 and presented in Figure 1.

To investigate the effects of different LSSs on TLS measurements, a special flat test board was prepared. Specimens of LSSs in the form of squares (0.15 m × 0.15 m) were glued to the surface of the board. The minimum square size was applied owing to the significant cost of the LSS specimens and their limited availability. Figure 1 shows the prepared targets (photos in daylight), raw piles (photos in artificial indoor lighting), and microscopic views of each LSS.

For more reliable and effective tests, three available scanners created by different manufacturers were used. Two of the scanners used (Z+F IMAGER 5016, Wangen im Allgäu, Germany, and FARO Focus^3D^, Lake Mary, FL, USA) are based on the principle of PS distance measurement. The third scanner (Leica ScanStation C10, Sankt Gallen, Switzerland) is based on the principle of TOF distance measurement. The two types of scanners (TOF and PS) have slightly different technical parameters and can be used for multiple purposes. TOF TLS is typically characterised by a longer maximum scanning distance (up to several kilometres) than PS TLS. TOF TLS is also characterised by a slightly lower precision of distance measurements and a significantly slower data acquisition rate than PS TLS [36]. Generally, PS scanners are suitable for various civil engineering purposes such as building information modelling [37], assessing architectural heritage [38], and deformation analysis of buildings and structures [39]. TOF scanners are convenient for most long-distance measurements, such as creating high-resolution digital terrain models [40], mine and cave surveys [41], and forestry inventories [42]. TOF scanners are also useful for short-distance civil engineering measurements, similar to PS scanners [43]. In practice, both types of scanners are often used interchangeably for multiple applications. The technical specifications of the scanners are listed in Table 2.

## 3. Tests and Calculations

**Figure 2:**
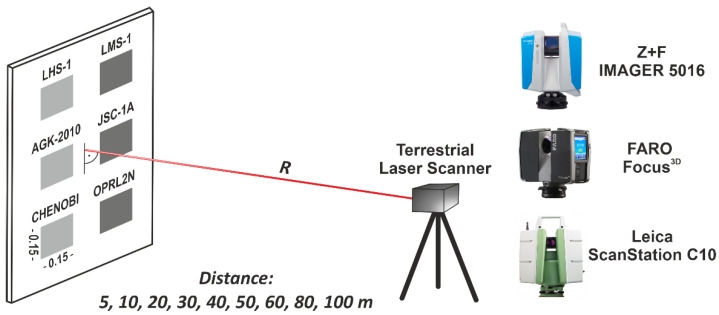
Setup of TLS measurements.

**Figure 3:**
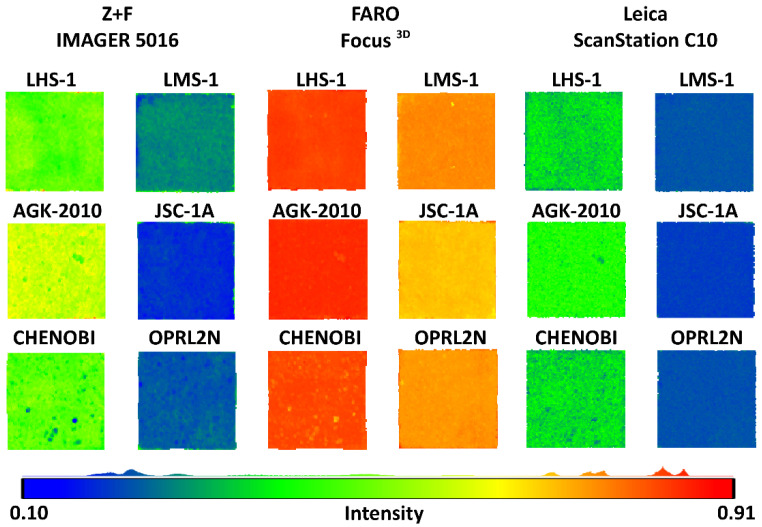
Point cloud intensities collected by TLS of LSS targets at a distance of 5 m.

This study conducted laser scanning of LSS specimens in an outdoor environment (temp. +10 °C ± 1 °C; r.h. 50% ± 1%). Specimens were scanned at nine different distances (5, 10, 20, 30, 40, 50, 60, 80, and 100 m). These distances were selected based on the authors’ previous experience with the scanners [19,44] and LSSs [29,30]. A TLS measurement from a distance of up to 100 m is sufficient for the construction of future habitats and structures on the Moon. During the measurements, the laser beam struck the LSS specimens perpendicularly. The incidence angle of the laser beam ranged from 0° to 2° and did not affect the values of the *intensity* (see Equation (1)). A schematic of the measurement setup is shown in Figure 2. As an example, the full results of the LSS measurements from a distance of 5 m are presented in Figure 3.

One of the key factors in laser measurements is the ability of the scanned material to reflect the laser beam. However, not every surface has this capability. Measurements can be conducted on porous surfaces, where Lambertian scattering occurs (called diffuse reflection). According to the Rayleigh and Fraunhofer criteria, diffuse reflection occurs when the surface roughness is greater than the wavelength of the incident radiation [45]. Measurements of smooth shiny surfaces (e.g., mirrors and polished metal) cannot be obtained by TLS owing to the law of reflection. The angle of incidence is equal to the angle of reflection, and no signal will be returned to the scanner. This type of reflection is described as specular reflection. In reality, most materials on Earth, such as soil, sand, concrete, ceramics, and wood, exhibit diffuse reflection (or a combination of diffuse and specular reflection), enabling TLS measurements. The equation defining the relationship between the transmitted signal power (*P_T_*) and the received signal power (*P_R_*) in laser scanners is expressed as follows [46]:

(1) $$intensity=\frac{P_{R}}{P_{T}}=\frac{\pi \rho \;cos\left(\Theta \right)}{4R^{2}}\eta_{Atm}\eta_{Sys}.$$

The returning energy of a laser beam is affected by the following properties: the target reflectivity of the scanned surface (*ρ*), the angle of incidence (*Θ*), the distance between the TLS scanner and target (*R*), the atmospheric transmission factor (*η_Atm_*), and the system transmission factor (*η_Sys_*). During the tests, the properties associated with the reflectivity of the LSS targets were analysed.

**Figure 4:**
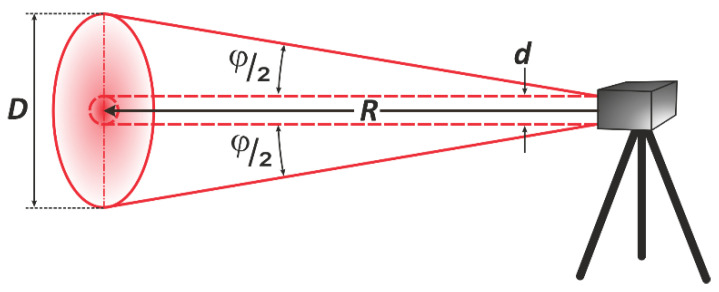
Footprint created by a laser beam.

To post-process the point clouds, it was necessary to determine the appropriate tested area for each LSS specimen. In this study, it is important to note that the laser beam is divergent. Thus, the area of a tested specimen is “effective” when the entire laser spot hits it during the measurement. The spot size of the laser beam is a function of the beam waist on the expander (*d*), beam divergence (φ), and distance (*R*). The spread of the laser beam emitted by the laser transmitter is shown in Figure 4. The theoretical laser beam diameter (*D*) and area (*A*) covered by a beam when it reaches a target can be calculated using Equations (2) and (3), respectively [47].

(2) D=φ·R+d

(3) $$A={\frac{\pi \left(\varphi \cdot R+d\right)}{4}}^{2}$$

During TLS measurements, the spot of the laser beam has different sizes, depending on the distance. The three scanners used were characterised by different laser beam divergences. Based on Equations (2) and (3), the theoretical diameter and area of the laser spots for all three scanners were established (see Table 2). The Z+F IMAGER 5016 scanner was characterised by the largest divergence. Thus, this was the least-favourable situation, and further calculations were conducted for this scanner. It should be mentioned that some TLS manufacturers use special lenses for collimation of the laser beam [48]. In this case, the laser beam is collimated by an expander (such as a reverse telescope) and transmitted to the object. The focused beam first narrows for a certain distance and then expands [49]. This approach creates a relatively small laser footprint at a distance of several dozen metres. The Leica ScanStation C10 uses this approach (see Table 2). Table 3 presents an overview of the diameter and area of the laser spot depending on the distance for the Z+F IMAGER 5016 scanner.

**Figure 5:**
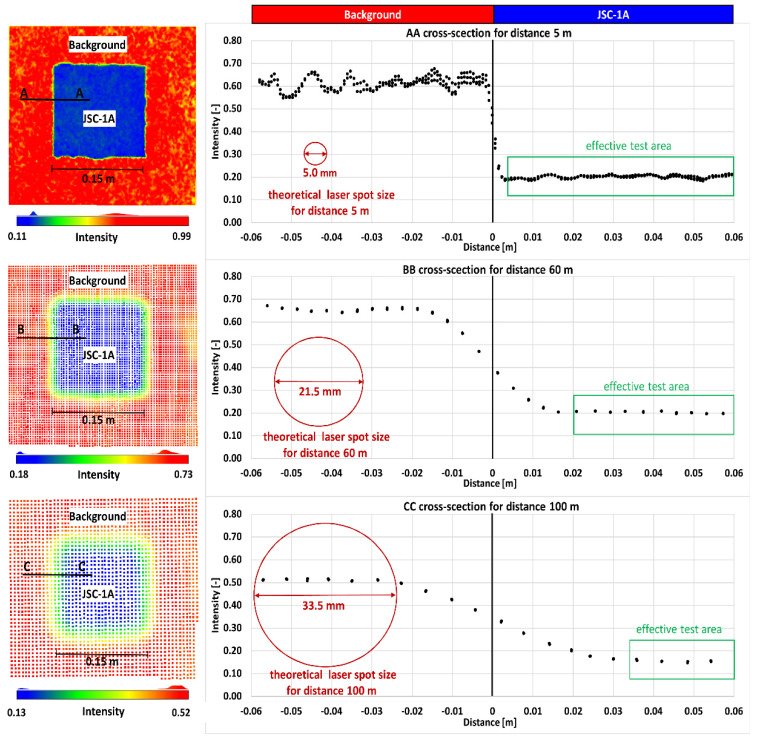
Effect of the spot size on the boundary measurement between two surfaces.

The size of the laser spot affects the quality and precision of the measurement. A situation often occurs in which the footprint of a laser beam on a surface covers an area including two or more objects [48]. These objects can be in one plane or at different distances. The reflected energy cannot be associated with one of these objects, but is interpreted as a weighted average value. Two objects with different surface properties typically exhibit dissimilar reflective properties. An object comprising a larger area of the laser footprint will have a larger effect on the laser beam energy returned. To determine the effective LSS area of the tested samples, the point clouds from JSC-1A target measurements were analysed in detail. Additionally, a detailed analysis was undertaken by creating cross-sections from narrow strips 0.008 m wide across the plate (called the background) and the JSC-1A specimen. In Figure 5, the measurement results for the three distances of 5, 60, and 100 m are presented. By analysing Figure 5, the effective area of the LSS specimen can be easily determined. It was decided to use the LSS area in which the entire laser spot hits the desired surface. The effective area of the LSS varied at different distances. The smallest effective area of an LSS specimen occurred at a distance of 100 m. For this case, the effective area was approximately 0.035 m based on the boundary between the background and JSC-1A specimen. Therefore, the same square area equal to 0.08 m × 0.08 m was adopted for all samples.

## 4. Results

**Figure 6:**
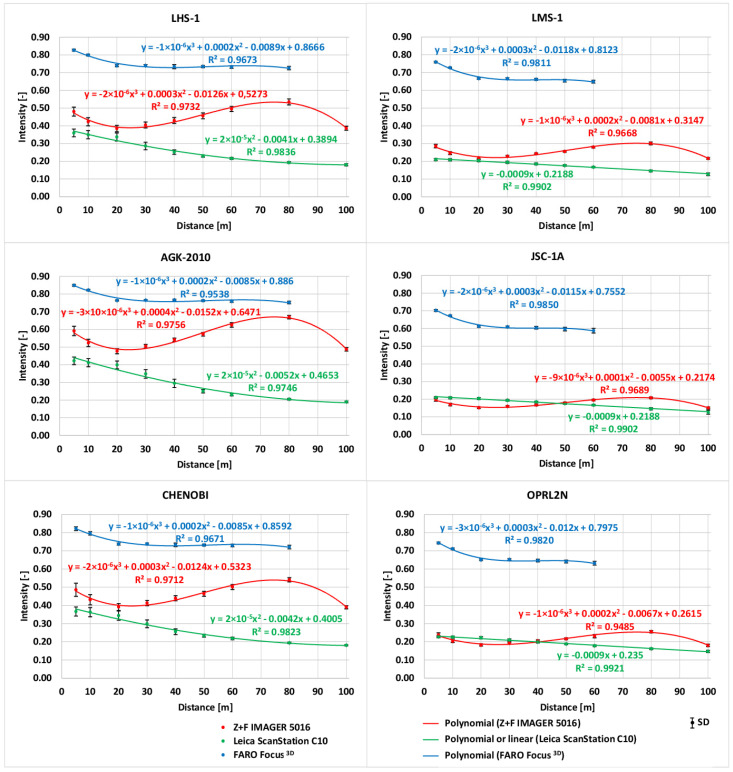
Relationship between the *intensity* and distance for different LSS specimens.

A total of nine measurements of six LSS specimens were performed using three scanners. In the case of the FARO Focus^3D^ scanner from a distance of 100 m, no point clouds were recorded for any of the LSS specimens. From a distance of 80 m, the scanner did not register point clouds for three of the LSS specimens (LMS-1, JSC-1A, and OPRL2N). The recorded data were processed using the CloudCompare software (cloudcompare.org) to analyse the *intensity* values. The collected *intensity* datasets of the LSS specimens were statistically analysed and used to create *intensity*–distance plots. In Figure 6, the mean values of the *intensity*, standard deviations, and fitted functions of the datasets are presented.

It is clear that the *intensity* values registered by the FARO Focus^3D^ are significantly higher than those registered by the Leica ScanStation C10 and Z+F IMAGER 5016. Nevertheless, some of the scans of the LSS specimens at distances of 80 and 100 m were not registered by the FARO Focus^3D^ scanner. It should be noted that this scanner had the shortest measuring distance of up to 120 m (see Table 2). For all of the LSS specimens scanned with the Leica ScanStation C10, the decrease in the *intensity* value is directly proportional to the increase in the measuring distance. This relationship can easily be described using a linear or quadratic polynomial function. In the case of the Z+F IMAGER 5016, the *intensity* value gradually decreases proportionally to the increase in the measuring distance, reaching minimum values at a distance of 20 m. For longer distances, the *intensity* value gradually increases proportionally to the increase in the measuring distance, reaching its peak at a distance of 80 m. The *intensity* values at a distance of 100 m are significantly lower than those obtained at 80 m. In the authors’ opinion, the scanner reached its registration capabilities at this distance. This relationship can be described using a cubic polynomial function. It is clear that the *intensity* values are much higher for LSS specimens that represent lunar highlands (LHS-1, AGK-2010, and CHENOBI) than those that represent lunar mare regions (LMS-1, JSC-1A, and OPRL2N). This means that the mare LSSs (characterised by a darker colour) absorb more of the laser beam energy. This phenomenon is especially visible for the Leica ScanStation C10 and Z+F IMAGER 5016.

**Figure 7:**
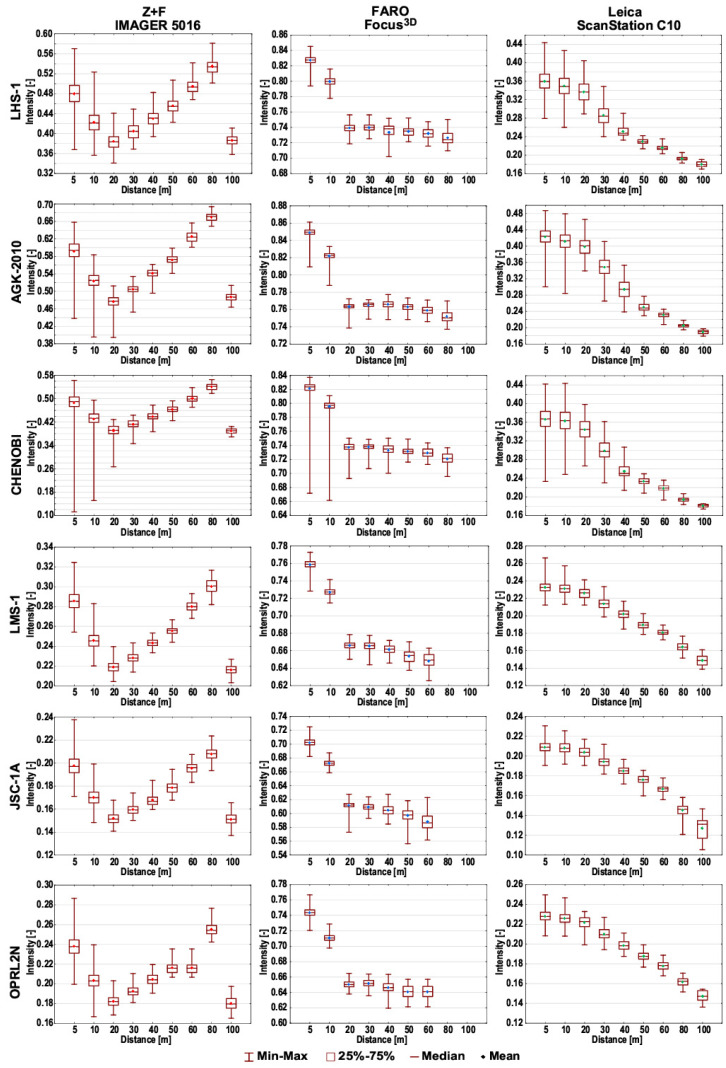
Box plots of the *intensity* data from TLS measurements.

The statistical characteristics of the acquired *intensity* results were considered a very important aspect of this research. Figure 7 presents a box plot with information regarding the minimum and maximum values, upper and lower quartiles, and median and mean values of the datasets. In the case of the Z+F IMAGER 5016, a large range (max–min) of *intensity* values is clearly noticeable for short measurement distances (5 and 10 m). This phenomenon is likely associated with the small laser spot size at these distances; thus, the apparatus can provide more detailed information. Such a relationship was not observed for the FARO Focus^3D^ scanner without considering the CHENOBI specimen. The range of *intensity* values for the CHENOBI specimen at short measurement distances is the largest of all the tested scanners. This specimen has a very different colour and grain size, which causes differences in the absorption and dispersion of the laser beam at each point. According to the technical specifications of the Leica ScanStation C10 scanner, the laser spot has a size of 7 mm for measurement distances of up to 50 m. The ranges of the *intensity* values for this scanner confirm the theoretical assumptions.

**Figure 8:**
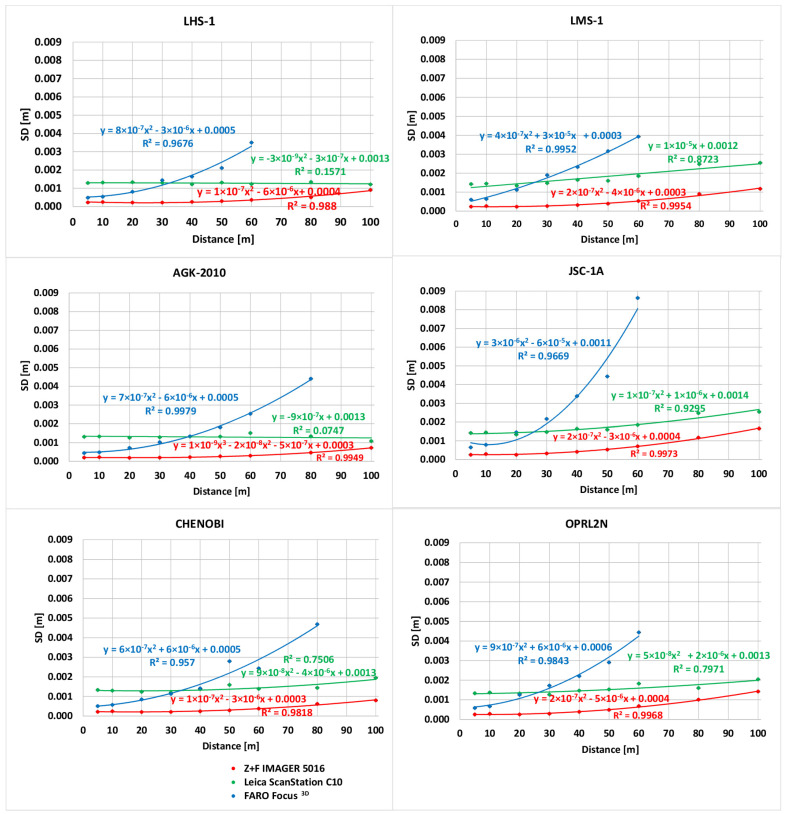
Error of TLS distance measurements for LSS targets.

Among the important parameters for determining whether TLS can be used for specific purposes is the precision and maximum feasible measurement distance. The type of scanned material affects the *intensity* value and, thus, affects the precision and maximum feasible distance of a measurement [50]. The effect of individual LSS specimens on the precision of TLS measurements conducted at various distances was investigated. The mean sum error (MSE) method presented by Chen [51] was used to determine the precision of the distance measurement for LSS targets. The LSS targets were assumed to be flat and homogeneous surfaces. The plane was fit based on the 3D coordinates of the points. The calculated distances, *d_i_*, of all points from the surface were considered as the real precision of the distance measurement. The computed standard deviation (SD) of *d_i_* was assumed to be the mean error of the distance measurements to the LSS targets. A complete analysis of the SD is presented in Figure 8. It is clear that the measurements conducted using the Z+F IMAGER 5016 scanner are characterised by the lowest error (SD) of the distance measurement. For highland LSSs (LHS-1, AGK-2010, and CHENOBI), the error of the distance measurement was less than one millimetre in all cases (regardless of the distance). For mare LSSs (LMS-1, JSC-1A, and OPRL2N), the error was slightly greater, but still did not exceed 2 mm. The error in the distance measured using the Leica ScanStation C10 scanner was slightly higher than that of the Z+F IMAGER 5016 scanner for highland LSSs (for all distances). The error ranged from 1 to 2 mm. Similar to the Z+F IMAGER 5016 scanner, the measurements conducted using the Leica ScanStation C10 scanner for mare LSSs were characterised by slightly larger errors in the distance measurement in comparison to the highland LSSs. The higher error in the distance measurement for the Leica ScanStation C10 scanner compared to the Z+F IMAGER 5016 scanner is understandable, as the two scanners use different types of rangefinders (TOF and PS, respectively). The results for the FARO Focus^3D^ scanner were the worst and were difficult to comprehend. The FARO Focus^3D^ scanner uses a PS rangefinder; therefore, it should have a distance measurement error similar to that of the Z+F IMAGER 5016 scanner. It should be noted that the maximum measurement distance of the FARO Focus^3D^ scanner is only 120 m. The increase in the error value of the distance measurement (relative to the increase in distance) was the most obvious for this scanner.

## 5. Discussion

The tests conducted in this study confirm that the use of TLS technology on the Moon is feasible. The FARO Focus^3D^ scanner and the other tested scanners performed satisfactorily up to distances of 50 m and 100 m, respectively. It is important to be aware of the technical limitations of TLS apparatuses that are constructed for measurements under Earth conditions of temperature, pressure, gravity, and radiation. Future TLS apparatuses prepared for lunar (or Martian) applications should be able to cope with extreme extra-terrestrial local conditions. The modification of terrestrial laser scanners for lunar applications will create a new type of apparatus, lunar laser scanners (LLSs). LLSs should have significantly more resistant construction than TLS instruments, especially in the case of low and high temperatures and space radiation. It should be noted that ordinary TLS scanners usually operate at ambient temperatures between −10 °C and +45 °C (see Table 2); thus, a special hermetic thermal casing will be required for LLSs. The lack of an atmosphere on the Moon will have a positive effect on the distance measurements of the rangefinder relative to Earth measurements; longer measuring ranges will be achieved on the Moon. The Earth’s atmosphere causes attenuation of the laser beam as it propagates through the atmosphere due to absorption, scattering, and other phenomena [52]. Electronic distance measurements by TLS or geodetic total stations on Earth usually include corrections for atmospheric parameters such as the temperature, pressure, and water vapour pressure [53,54]. On the Moon, corrections to atmospheric parameters will be significantly smaller or will not be necessary. It should be noted that TLS scanners can be equipped with additional sensors, e.g., digital cameras or thermal cameras, which would provide additional useful data for lunar applications. Other sensors, such as gyroscopes, compasses, or GPS, enable automatic registration of individual scans into a single point cloud. TOF and PS scanners have different advantages and disadvantages (considering the feasible maximum scanning distance, accuracy, and scan rate). For the most-effective lunar applications, future LLSs should be equipped with both types of rangefinders (TOF and PS). This would allow the operator (in situ or remotely) to choose one rangefinder for a specific task or use both rangefinders simultaneously.

**Figure 9:**
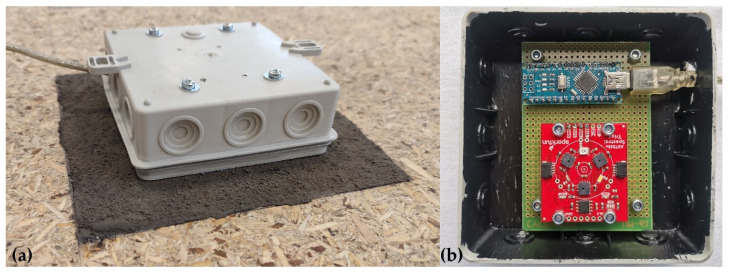
Spectrometer measurement of an LSS specimen (**a**) general view (**b**) interior of the device.

The investigation of the radiometric behaviour of LSSs in terrestrial laser scanner measurements is a first step toward their future utilisation on the Moon. In the authors’ opinion, future research should focus on the performance of TLS measurements associated with the erection of lunar habitats. Terrestrial laser scanners use lasers with different wavelengths; thus, the wavelength that is most appropriate for lunar applications should also be considered. A spectrometer can be used for this purpose, as a spectrometer can capture the *intensity* of the reflected light for a given wavelength. Currently, spectroscopy techniques are widely used for measuring the chemical and physical properties of materials, such as soil monitoring [55] and water stress in plants [56]. The device created by the research team for the future research consists of a SparkFun Triad Spectroscopy Sensor, Niwot, Colorado, USA (AS7265x consisting of three spectral sensors: AS72651 (UV), AS72652 (WHT), and AS72653 (IR)) and a Microcomputer Arduino Nano V3.0 ATMEGA328P (arduino.cc). It can detect light with wavelengths from 410 nm to 940 nm [57]. The sensor can provide information about how a surface absorbs and reflects light of different wavelengths (at 18 individual light frequencies). Each LSS was tested separately using the SparkFun. The sensor was placed face-down on each LSS target, and measurements were recorded during a period of 20 s. To eliminate the effects of ambient light, the AS7265x sensor was placed in a specially prepared protective shell (Figure 9a). In Figure 9b, the spectrometer devices during the measurement of a sample of the JSC-1A LSS are presented. Apart from the LSS specimens, white paper was also measured as a basic Lambert material. The raw measurement data for the LSS specimens were normalised with respect to the white paper.

**Figure 10:**
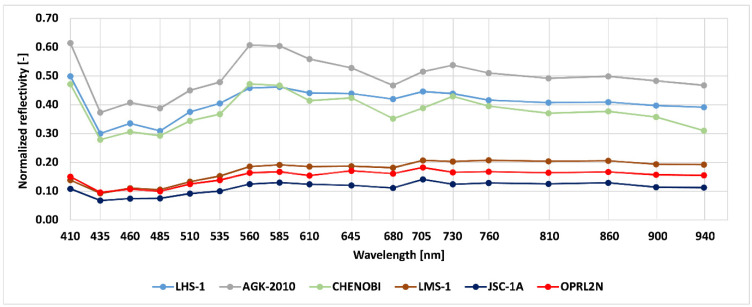
Normalised results of the LSS specimen targets for different wavelengths.

The normalised results (reflectivity of LSS/reflectivity of white paper) of the tested LSS specimens are shown in Figure 10. The reflectance values at wavelengths of 535 and 900 nm are of particular interest because this wavelength corresponds very closely to the Leica ScanStation C10 (532 nm) and FARO Focus^3D^ (905 nm) scanners. Unfortunately, the Z+F IMAGER 5016 scanner has a laser with a wavelength of 1500 nm, which exceeds the measuring scale of the SparkFun sensor.

In Figure 10, two distinct families of results associated with the mare and highland LSSs can be observed. The development process of both apparatuses and software dedicated to lunar applications should consider these phenomena. In general, it seems that rangefinders should be used from a wavelength of 560 nm.

The conducted research programme proved that TLS has the potential to become an important technology used in lunar civil engineering. Automatic and non-destructive analysis of lunar soil is crucial for any future construction effort on the Moon. TLS apart from traditional geodetic purposes can be used for the organizing of a lunar building site, assessment of the homogeneity of the lunar soil, quality control measurements of an ongoing construction process, and before the commissioning of a structure. Future applications may alco cover identification of places best for sourcing lunar aggregate (e.g., based on ilmenite) and establishing lunar open pit mines.

The analysis of the bearing capacity of lunar soil and its other geotechnical properties were out of the scope of the conducted research programme. Nevertheless, it is possible that the results of TLS scans would be useful for the assessment of the properties of lunar soil. To conduct such an analysis, multiple tests of the geotechnical properties of LSSs are needed. To replicate the lunar soil density and void ratio, one should scan (using TLS) lunar soil standing on the lunar surface. Only having the results of TLS conducted in low gravity and with the lack of an atmosphere, the research programme aiming to replicate lunar soil density and void ratio would be enabled.

## 6. Conclusions

The following conclusions were drawn from this research:

- It is possible to conduct measurements of LSSs using TLS; hence, future applications of TLS technology on the Moon are feasible.
- The LSS specimens representing lunar mare and highland regions are characterised by different degrees of laser beam absorption. The LSS specimens representing mares, due to their darker colour in comparison to the LSSs representing highlands, return less energy to the scanner. Therefore, the feasible TLS measurement distance for lunar mare regions will be shorter than that for lunar highland regions.
- Measurements conducted using the FARO Focus^3D^ scanner at distances of 100 m and 80 m were impossible to execute for all tested LSS specimens and mare LSS specimens, respectively.
- The Z+F IMAGER 5016 (PS) scanner was characterised by the highest precision of distance measurements. The Leica ScanStation C10 (TOF) scanner was characterised by slightly lower precision.
- Detailed studies focusing on the best wavelength for lunar rangefinders should be conducted.
- Significant modifications are required for the construction of scanners dedicated to lunar applications. Apart from the needs associated with extreme local conditions, wavelength issues are of special interest.
- To replicate the lunar soil density and void ratio, the results of TLS conducted on the lunar surface are needed.
- Combining multiple non-destructive technologies for lunar civil engineering, scanning should be enquired in future research programmes.

## Figures and Tables

**Table 1:** Lunar soil simulants used in this study.

Type	Acronym	Name	Availability	Country
Highlands	LHS-1	Lunar Highlands Simulant [32]	YES	USA
	AGK-2010	Lunar Soil Analog (Analog Gruntu Księżycowego—in Polish) [33]	NO	Poland
	CHENOBI	CHemically ENhanced OB-1 Lunar Highlands Regolith Physical Simulant [34]	NO	Canada
Mare	LMS-1	Lunar Mare Simulant [32]	YES	USA
	JSC-1A	Johnson Space Center [8]	NO	USA
	OPRL2N	Off Planet Research Mare Simulant [35]	YES	USA

**Table 2:** Basic characteristics of terrestrial laser scanners.

	Z+F IMAGER 5016	FARO Focus^3D^	Leica ScanStation C10
Type of rangefinder	PS	PS	TOF
Laser wavelength	1500 nm	905 nm	532 nm
Type of wavelength	Infrared	Near-infrared	Green
Max scan rate points/second	1,100,000	1,000,000	up to 50,000
Max measurement distance	365 m	120 m	300 m @ 90%
Distance measurement error	±1 mm + 10 ppm/m	±2 mm	±4 mm (1–50 m)
Beam divergence	0.3 mrad	0.19 mrad	–
Beam diameter	~3.5 mm at exit	3.0 mm at exit	0–50 m: 4.5 mm *, 7 mm **
Operating temperature	−10 °C … +45 °C	5 °C … +40 °C	0 °C … +40 °C
Field of view (h/v)	360°/320°	360°/305°	360°/270°
Additional sensors	HDR camera, optional IR camera, positioning system (barometer, acceleration sensor, gyroscope, compass, GPS)	Digital camera (70 megapixels), compass, height sensor (altimeter)	digital camera (4 megapixels)

* FWHH-based; ** Gaussian-based.

**Table 3:** Diameter and area of laser spots for Z+F IMAGER 5016.

*R* (m)	5	10	20	30	40	50	60	80	100
*D* (mm)	5.0	6.5	9.5	12.5	15.5	18.5	21.5	27.5	33.5
*A* (mm^2^)	19.6	33.2	70.9	122.7	188.7	268.8	363.1	594.0	881.4

## Data Availability

The data presented in this study are available upon request from the corresponding author.
